# Clinical characteristics and severity of hand, foot, and mouth disease by virus serotype: A prospective hospital-based cohort study

**DOI:** 10.1371/journal.pntd.0013039

**Published:** 2025-05-23

**Authors:** Yu Li, Junmei Yang, Lu Liang, Kai Wang, Lance Turtle, Peng Li, Yonghong Zhou, Yuanfang Shen, Peng Cui, Chongchen Zhou, Qi Qiu, Chun Guo, Mengyao Zeng, Lu Long, Tianchen Zhang, Malik Peiris, Benjamin J. Cowling, Tom Solomon, Yibing Cheng, Peng Wu, Hongjie Yu

**Affiliations:** 1 School of Population Medicine and Public Health, Public Health Emergency Management Innovation Center, Chinese Academy of Medical Sciences & Peking Union Medical College, Beijing, China; 2 WHO Collaborating Centre for Infectious Disease Epidemiology and Control, School of Public Health, Li Ka Shing Faculty of Medicine, The University of Hong Kong, Hong Kong, China; 3 Children’s Hospital Affiliated to Zhengzhou University, Henan Children’s Hospital, Zhengzhou, China; 4 Department of Medical Records & Statistics, Sichuan Provincial People’s Hospital, School of Medicine, University of Electronic Science and Technology of China, Chengdu, China,; 5 West China School of Public Health and West China Fourth Hospital, Sichuan University, Chengdu, China; 6 Shanghai Institute of Infectious Disease and Biosecurity, School of Public Health, Fudan University, Shanghai, China; 7 NIHR Health Protection Research Unit for Emerging and Zoonotic Infections, University of Liverpool, Liverpool, United Kingdom; 8 Tropical & Infectious Disease Unit, Royal Liverpool University Hospital (Member of Liverpool Health Partners), Liverpool, United Kingdom; 9 School of Public Health, Huazhong University of Science and Technology, Wuhan, China; 10 NHC key Lab of Reproduction Regulation (Shanghai Institute of Planned Parenthood Research), Medical School, Fudan University, Shanghai, China; 11 Division of Infectious Disease, Key Laboratory of Surveillance and Early-warning on Infectious Disease, Chinese Center for Disease Control and Prevention, Beijing, China; 12 The Walton Centre NHS Foundation Trust, Liverpool, United Kingdom,; 13 Fudan University, Key Laboratory of Public Health Safety, Ministry of Education, Shanghai, China; University of Malaya Faculty of Medicine, MALAYSIA

## Abstract

The circulating enteroviruses (EVs) serotypes in hand, foot and mouth disease (HFMD) inpatients remained unclear. This study aimed to investigate the serotype-specific associations between clinical characteristics and severity of HFMD inpatients. The study utilised a prospective, hospital-based cohort design and a tiered diagnostic algorithm incorporating real-time RT-PCR and nested RT-PCR for serotyping. Clinical data were prospectively collected throughout hospitalization. Clinical severity was measured using diagnoses of central nervous system (CNS) complications and three other outcomes. A total of 1768 inpatients were enrolled consecutively between February 2017 and February 2018. The proportions of CNS complications varied by serotype (p < 0.001), with the highest for EV-A71 (40%), followed by CV-A4 (17%), CV-A2 (13%), CV-A10 (10%), CV-A6 (7%), and CV-A16 (4%). Children with CV-A2 and CV-A4 were less likely to have rashes on hands, feet, or buttocks and more likely to develop high fever, while those with EV-A71 had fewer mouth lesions. Of 230 lab-confirmed HFMD inpatients with CNS complications, EV-A71 accounted for 45% while CV-A6, CV-A16, CV-A4, CV-A10 and CV-A2 accounted for 35%. The logistic regression analysis revealed that non-CNS-specific symptoms such as cold limbs and vomiting, and clinical testing indicators including blood globulin, platelet, serum chloride and neutrophil counts, were associated with CNS complications. Non-EV-A71 EVs can also cause severe diseases, but those with EV-A71 infection are more likely to suffer CNS complications and other severe manifestations. The study highlighted the emergence of enterovirus serotypes, suggesting the need for future research on virus changes and associated disease burden.

## Author summary

Hand, Foot, and Mouth Disease (HFMD) is a common viral childhood illness with more than 1 million cases reported annually in China. It is caused by enteroviruses including EV-A71, coxsackieviruses, etc. While HFMD is mostly self-limited, it can lead to severe or even fatal complications in a minority of patients. Our prospective study in a tertiary children's hospital in central China demonstrated that over half of inpatient HFMD cases with CNS complications were caused by non-EV-A71 enteroviruses, but children with non-EV-A71 enteroviruses were less likely to develop clinical severe cases compared with EV-A71. Children with CV-A2 and CV-A4 were less likely to have rashes on hands, feet, or buttocks and more likely to develop high fever, while those with EV-A71 were less likely to have lesions in mouths. Our study identified several non-CNS-specific symptoms (cold limbs and vomiting) and routine clinical tests (blood globulin, platelet, serum chloride and neutrophil counts), which can be used to alert clinicians of CNS complications. Among children with HFMD, non-EV-A71 EVs can also cause severe diseases, but those with EV-A71 infection are more likely to suffer CNS complications and other severe manifestations. Continuous surveillance of emerging enteroviruses and their clinical severities is essential.

## Introduction

Hand, foot and mouth disease (HFMD) is a common infectious disease among children [1]. Repeated HFMD epidemics have occurred in the Asia-Pacific region in recent decades, posing a significant threat to children’s health [1]. Causative agents of HFMD are mainly human enteroviruses (EVs) of Species A. Among all enterovirus serotypes related to HFMD, EV-A71 has been the most widely studied because it is thought to be the most severe, being responsible for the majority of fatal HFMD cases [1]. In the past decade there have also been a number of reports on HFMD associated with CV-A6, CV-A16 and CV-A10 [2–4]. However, non-EV-A71 EVs were rarely systematically characterized on clinical manifestations and clinical severity in a single large study including multiple serotypes of EVs. In prior studies, various clinical tests have been investigated as potential biomarkers for predicting severity, but the strength of associations has rarely been compared between different tests. Therefore, we sought to systematically compare the clinical features and severities of a wide range of enteroviruses serotypes in children with HFMD. Also, we described symptoms and tests with the potential for predicting severe cases.

## Methods

### Ethics

The study protocol was reviewed by the Institutional Review Boards of Henan Children’s Hospital (IRB#YZ-17–006), Chinese Centre for Disease Prevention and Control (IRB#201624), and Public Health School of Fudan University (IRB#2017-12-0654). Written informed consent was provided by parents or legal guardians of study participants on enrollment.

### Study design, setting, and participants

Henan Children’s Hospital is a top-tier pediatric hospital in Zhengzhou, Henan province, in central China. All inpatient HFMD cases with clinical diagnosis of HFMD admitted to department of infectious disease or pediatric Intensive Care Unit in Henan Children’s Hospital were invited to participate in this study from February 15, 2017 to February 15, 2018. Patients who were at the convalescent stage of HFMD, those discharged within 24 hours, or those whose parents refused to participate were excluded. HFMD was suspected if children presented with papulovesicular/maculopapular rash on the hands, feet, mouth or buttocks, with or without vesicles/ulcers in the mouth and fever [5]. After written informed consent was obtained from the guardian of the participant, patient data including clinical manifestations, routine laboratory test results, imaging examinations, diagnoses, treatment, medical history and demographics were collected by trained research staff using structured questionnaires on a daily basis throughout hospitalization.

Throat and rectal swabs were collected from all enrolled cases within 48 hours of admission, which were in addition to the routinely collected diagnostic samples, while stool samples were collected as part of routine care as soon as possible after admission. The collected swabs were put into a 15 ml conical tube containing 3.5 mL of UTM viral transport medium (Yocon, Beijing, China) while stool samples were placed in conical plastic tubes, and all samples were stored at -80 °C until testing. In order to illustrate the circulating enteroviruses among clinically milder HFMD cases, up to three outpatient cases were consecutively enrolled every other day for collecting throat swabs and rectal swabs, and brief clinical data, details of which have been described elsewhere [6].

### Clinical severity definition

The clinical severity was measured using the following four indicators. The first indicator was central nervous system (CNS) complications, defined as encephalitis, brainstem encephalitis, encephalomyelitis, acute flaccid paralysis, meningitis, and other severe CNS syndromic presentations, following World Health Organization criteria [7]. The second was the requirement for supportive treatment, including systemic corticosteroids or intravenous immunoglobulin (IVIG) or mechanical ventilation or inotropic agents [5]. The third was ICU admission during hospitalization. The fourth indicator was the length of hospital stay (LOS) over 5 days. The primary outcome measure for clinical severity was the diagnosis of CNS complications, while the other three were used as the secondary outcomes. HFMD cases were classified as severe if they met at least one of the four predefined clinical indicators.

### Virological testing

For the enrolled inpatient HFMD cases, throat swabs, stool samples, rectal swabs and other samples were selected in turn for virological testing until the specific enteroviruses were identified at the Fudan University biosafety level 2 laboratory (Shanghai, China), while for the enrolled outpatient HFMD cases, only throat swabs were tested. The laboratory adopted a stepwise approach to diagnostic testing composed of real-time RT-PCR and several nested RT-PCRs, with details previously described elsewhere [8]. For inpatient HFMD cases whose testing results were negative for enteroviruses or had no specimens tested in our laboratory, we further cross-checked their diagnostic testing results at the hospital laboratory which used a commercial one-step multiplex RT-PCR kit (EV-A71, CV-A16 and other enterovirus Viral RNA Qualitative Diagnostic Kit (PCR Fluorescence Probing), Mole Bioscience, China, patent number: 20143402367), with additional details in S1 Text.

### Statistical analyses

Analyses of clinical manifestations were restricted to HFMD inpatient cases as the clinical information collected for outpatient cases was limited and the sampling method was different from that of inpatient cases. Clinical manifestations of outpatient cases were described elsewhere [6]. Laboratory confirmed enterovirus infected HFMD cases only were included in the analysis of clinical manifestations, excluding those without any clinical specimens collected, and those where PCR results were negative for enteroviruses. For analyses studying the relationship between serotype and clinical severity, only those cases where an enterovirus serotype could be identified, and in greater than 20 cases, were included. The associations of clinical tests on admission with the presence of CNS complications were analyzed for all laboratory confirmed HFMD inpatient cases. The clinical tests analysed included complete blood count (white blood cell, neutrophil, lymphocyte, monocyte, eosinophil and platelet counts), neurological injury tests (S100 protein and neuron−specific enolase), myocardial enzymes (creatine kinase MB and alanine transferase), electrolytes (serum sodium and serum chloride), blood proteins (total protein, globulin, C-reactive protein) and blood glucose. We calculated the neutrophil to lymphocyte ratio by dividing neutrophil count by lymphocyte count from the same blood count measurement and included this variable in multivariate analyses.

Categorical variables between differing groups were compared using the chi-square test or Fisher’s exact test as appropriate. Wilcoxon Rank Sum test or Kruskal-Wallis test was used for assessing the difference in the continuous variables among different groups. P < 0.05 was considered as statistically significant, except when comparing characteristics between pairs of serotypes, where the Bonferroni correction applied. Logistic regression models were used to calculate the adjusted odds ratio (aOR) of CNS complications associated with clinical symptoms and clinical testing indicators, controlling for age, enterovirus serotype, preterm birth, low birth weight and underlying medical conditions. These confounders were selected based on biological and clinical plausibility. In the analyses using logistic regression models, values of clinical testing indicators were standardized through subtracting means and then divided by standard deviations, in order to control the effects of differing units and scales of different tests.

## Results

### Participants enrollment and etiology

There were 2562 children admitted with HFMD during the study period of 15 February 2017 through 15 February 2018, of whom 2524 were eligible for enrollment and 1840 (73%) agreed to participate (S1 and S2 Figs). The characteristics of the cases declining to participate in the study were not remarkably different from the participants (S1 Table), except that more ICU admissions were observed in participants (p < 0.0001). Among the 1840 HFMD inpatient cases recruited, 1768 (96%) were laboratory confirmed cases. CV-A6 (31%), CV-A16 (19%), EV-A71 (15%), CV-A10 (5%), CV-A4 (3%) and CV-A2 (3%) accounted for 76% of laboratory confirmed inpatient cases. For the remaining 427 laboratory confirmed inpatient cases (24%), specific enterovirus serotypes were determined for 54 cases, while the remaining 373 cases were undetermined enterovirus serotypes (S2 Fig). The proportions of various enterovirus serotypes were significantly different between inpatient and outpatient HFMD cases (p < 0.0001), with lower proportions of EV-A71 (7%) and higher proportions of CV-A6 (42%) and CV-A16 (35%) among outpatient cases (S3 Fig). Among 1768 lab-confirmed inpatient cases, 266 (15%) cases received EV-A71 vaccines, while among 258 EV-A71 infected inpatient cases, only 10 (4%) cases were vaccinated.

### Participants demographics

The age was significantly different among different serotypes with EV-A71 affecting older children, and 56% of inpatient HFMD cases with EV-A71 were two years and above whereas more than half of those with other serotypes were younger than two years (Table 1). There was no significant difference across serotypes in distribution of sex, underlying medical conditions, preterm births, or low birth weight. The median and interquartile range (IQR) of intervals between illness onset and admission were 2 (1–3) days for laboratory confirmed HFMD inpatient cases, of which the difference was statistically significant among different serotypes (p < 0.0001), with the longest interval for EV-A71 (S4 Fig). Of the enrolled laboratory confirmed inpatient HFMD cases, two cases eventually died, one was with EV-A71 and the other one was with CV-B2. All other surviving patients were discharged home, except for one patient who was transferred to another hospital.

**Table 1:** Characteristics of laboratory confirmed inpatient HFMD cases by virus serotype at the Children’s Hospital, February 2017–Februray 2018.

Characteristics	Total (N = 1768)	EV-A71 (N = 258)	CV-A4 (N = 59)	CV-A2 (N = 48)	CV-A10 (N = 79)	CV-A6 (N = 554)	CV-A16 (N = 343)	Other (N = 427)	P value
Age, years, median [IQR]	1.7 [1.2, 2.8]	2.2 [1.4, 3.4]	1.7 [1.3, 2.7]	1.5 [1.2, 2.3]	1.6 [1.2, 2.3]	1.4 [1.1, 2.3]	1.9 [1.4, 2.9]	1.6 [1.1, 2.7]	<0.0001
Age group									
0-6 months	21 (1)	4 (2)	0 (0)	0 (0)	0 (0)	4 (1)	1 (0)	12 (3)	0.0005
7-11 months	241 (14)	19 (7)	4 (7)	5 (10)	12 (15)	100 (18)	35 (10)	66 (15)	
1 years	809 (46)	90 (35)	35 (59)	27 (56)	42 (53)	281 (51)	148 (43)	186 (44)	
2-14 years	697 (39)	145 (56)	20 (34)	16 (33)	25 (32)	169 (31)	159 (46)	163 (38)	
Male	1121 (63)	164 (64)	40 (68)	35 (73)	50 (63)	341 (62)	227 (66)	264 (62)	0.57
Underlying medical conditions ^*^	28 (2)	5 (2)	2 (3)	0 (0)	2 (3)	8 (1)	5 (1)	6 (1)	0.75
Pre-term birth^†^	87 (5)	16 (6)	2 (3)	4 (8)	6 (8)	18 (3)	19 (6)	22 (5)	0.30
Low birth weight^‡^	54 (3)	10 (4)	2 (3)	1 (2)	3 (4)	15 (3)	13 (4)	10 (2)	0.87

Data are n (%) or median (IQR). ^*^ Underlying medical conditions included epilepsy (5), rickets (5), brain damage (3), adenoid hypertrophy (3), cranial nerve injury (3), developmental delay (2), progressive muscular dystrophy (2), anaemia (2), aniridia (1), renal fusion (1), tuberous sclerosis (1). ^†^ Defined as birth of a baby occurred before 37 weeks of gestation. ^‡^ Defined as birth weight lower than 2500 g.

### Clinical characteristics

**Fig 1:**
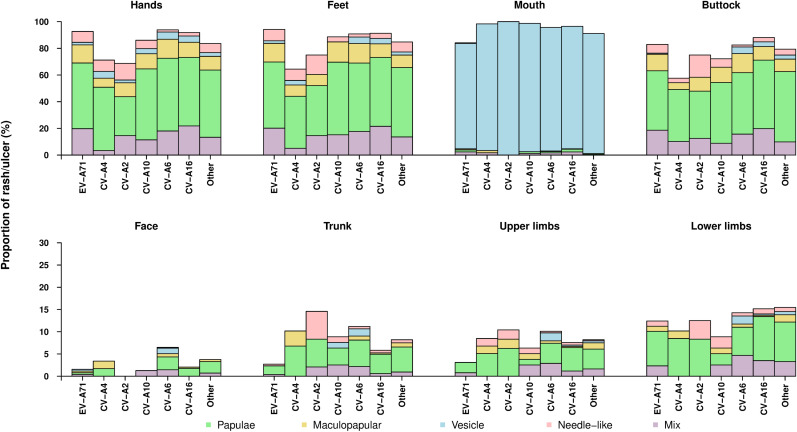
Proportions of HFMD inpatient cases presenting with different types of rashes on different body parts, stratified by enterovirus serotype, at the Children’s hospital from February 2017 to February 2018.

The distribution of rash among different parts of the body varied significantly according to serotype. Rash was most frequently observed on the hands, feet, buttocks and in the mouth (Fig 1). Rash on the hands (p < 0.001), feet (p < 0.001) and buttocks (p < 0.0001), was observed less frequently for children with CV-A2 or CV-A4 infection compared with other serotypes. Oral lesions were less frequently observed in those with EV-A71 infection (p < 0.001) than with other serotypes. The proportions of cases with rashes on other parts of the body were less than 20%, but also varied significantly with infecting serotype except on the lower limbs (p = 0.84). 97% of laboratory confirmed inpatient cases presented with fever. The peak temperatures of cases with fever varied statistically significantly among different serotypes (p < 0.001), which were the highest for children with CV-A2 and the lowest for those with CV-A16 (S5 Fig).

### Clinical severity by virus serotype

**Fig 2:**
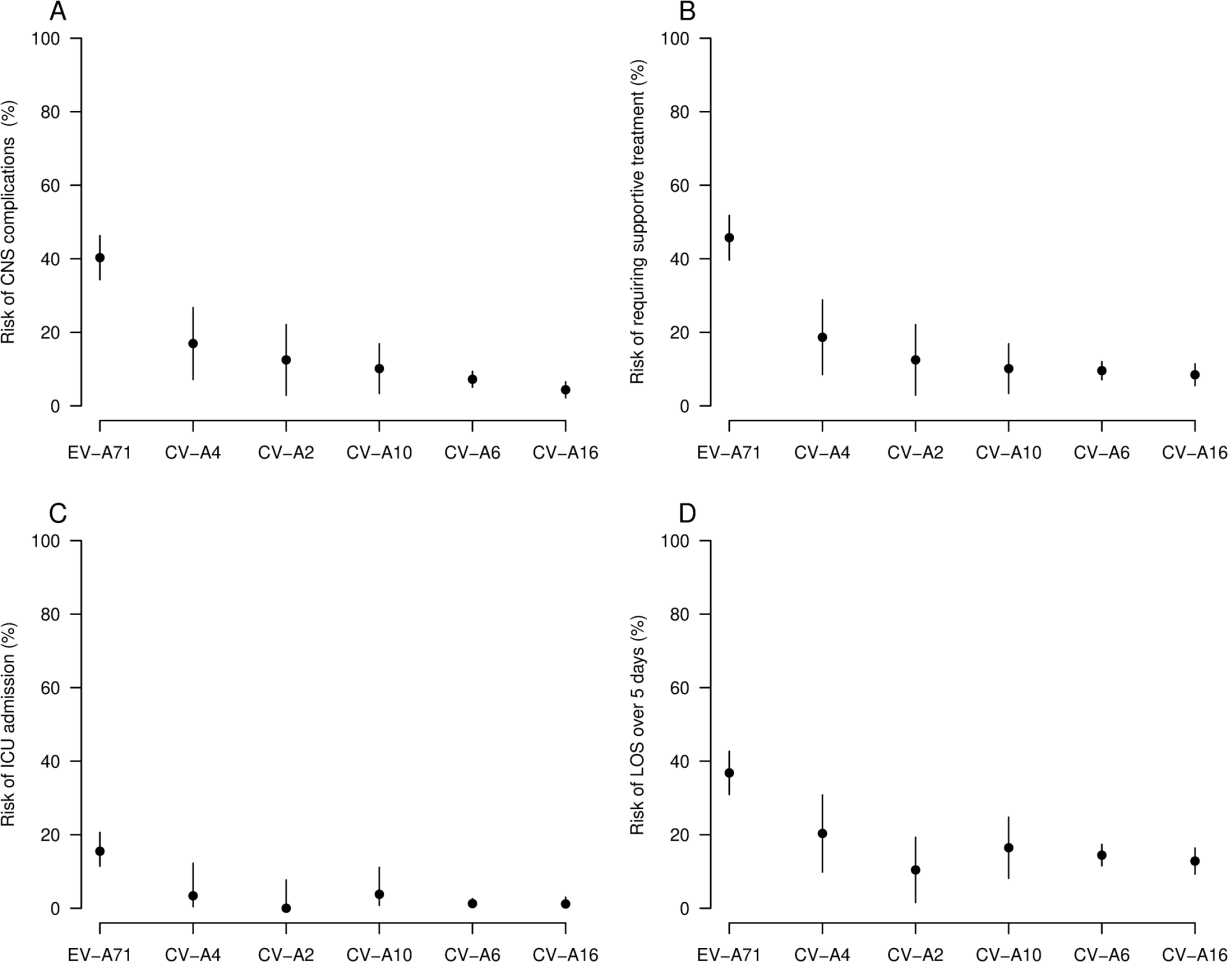
The risk of having one of the four predefined clinical outcomes by virus serotype among HFMD inpatient cases at the children’s hospital, February 2017-Februray 2018. A) Diagnoses as CNS complications. B) Requiring supportive treatment, including systemic corticosteroids and/or IVIG and/or mechanical ventilation and/or inotropic agents. C) ICU admission. D) LOS over 5 days.

The proportions of cases diagnosed with CNS complications varied across different infecting serotypes (Fig 2), with the highest proportion for EV-A71 (40%), followed by CV-A4 (17%), CV-A2 (13%), CV-A10 (10%), CV-A6 (7%), the lowest proportion for CV-A16 (4%). This difference was statistically significant (p < 0.001). Furthermore, pairwise comparisons for serotypes demonstrated CNS complications were consistently more likely with EV-A71 infection than any other serotype, and that this difference was statistically significant. The only other significant difference observed was that CV-A4 was more likely to result in neurological disease than CV-A16 (p < 0.001). EV-A71 also caused significantly worse disease than the other serotypes as measured by three other indicators of clinical severity: need for supportive treatment, ICU admission and longer LOS (Fig 2). All other serotypes caused similar levels of severe disease by these measures.

### Clinical characteristics of cases with CNS complications

230 (13%) of lab-confirmed inpatient HFMD cases were diagnosed with CNS complications, of which EV-A71 accounted for 45% while CV-A6 (17%), CV-A16 (7%), CV-A4 (4%), CV-A10 (3%) and CV-A2 (3%) accounted for 34%. Brainstem encephalitis was the most frequent CNS complication for all serotypes, except CV-A4 (Table 2). Most neurological findings were more frequent in EV-A71 cases of CNS disease, except for convulsions which were observed more frequently for other serotypes. The proportions of patients with both fever duration ≥3 days and reduced motor activity were relatively high for EV-A71. We observed four patients with cardiorespiratory failure, pulmonary edema and pulmonary hemorrhage following CNS involvement.

**Table 2:** Clinical manifestations of HFMD cases with CNS complications by virus serotype.

Clinical diagnoses and manifestations	EV-A71 (N = 104)	CV-A4 (N = 10)	CV-A2 (N = 6)	CV-A10 (N = 8)	CV-A6 (N = 40)	CV-A16 (N = 15)	P value
**CNS complication categories** ^*^							
Brainstem encephalitis	74 (71)	4 (40)	6 (100)	6 (75)	31 (78)	12 (80)	0.12
Encephalitis	14 (13)	5 (50)	0 (0)	2 (25)	5 (12)	1 (7)	0.055
Encephalomyelitis	12 (12)	0 (0)	0 (0)	0 (0)	3 (8)	0 (0)	0.71
Meningitis	1 (1)	0 (0)	0 (0)	0 (0)	1 (2)	2 (13)	0.13
Acute flaccid paralysis	1 (1)	0 (0)	0 (0)	0 (0)	0 (0)	0 (0)	1.00
Epilepsy seizure	2 (2)	1 (10)	0 (0)	0 (0)	0 (0)	0 (0)	0.38
**Systemic**							
Fever duration ≥ 3 days	96 (92)	7 (70)	1 (17)	3 (38)	17 (42)	8 (53)	<0.0001
Peak temperature ≥ 38.5°C	95 (91)	8 (80)	6 (100)	8 (100)	37 (92)	11 (73)	0.20
Reduced motor activity	94 (90)	5 (50)	2 (33)	6 (75)	26 (65)	11 (73)	0.0001
**Cutaneous**							
Skin rash	103 (99)	10 (100)	5 (83)	8 (100)	39 (98)	15 (100)	0.18
Oral lesion	96 (92)	10 (100)	6 (100)	8 (100)	39 (98)	15 (100)	0.81
**Neurological**							
Time from illness onset to neurological onset, days	1 (0, 2)	0 (0, 1)	0 (0, 2)	0 (0, 1)	0 (0, 1)	0 (0, 2)	0.066
Shiver	91 (88)	6 (60)	5 (83)	4 (50)	31 (78)	13 (87)	0.039
Limb jitter	46 (44)	1 (10)	0 (0)	4 (50)	6 (15)	4 (27)	0.003
Irritability	42 (40)	1 (10)	3 (50)	2 (25)	13 (32)	5 (33)	0.42
Startled response	44 (42)	2 (20)	1 (17)	2 (25)	7 (18)	7 (47)	0.051
Convulsion	7 (7)	6 (60)	2 (33)	2 (25)	10 (25)	2 (13)	0.0001
Altered consciousness	44 (42)	2 (20)	0 (0)	1 (12)	4 (10)	3 (20)	0.001
Limb weakness	29 (28)	1 (10)	0 (0)	0 (0)	3 (8)	1 (7)	0.014
Ataxia	17 (16)	1 (10)	0 (0)	1 (12)	3 (8)	2 (13)	0.79
Other neurological symptoms^†^	21 (20)	0 (0)	0 (0)	0 (0)	1 (2)	0 (0)	0.016
**Respiratory**							
Tachypnea	19 (18)	0 (0)	0 (0)	1 (12)	1 (2)	0 (0)	0.042
Altered respiratory rhythm	29 (28)	1 (10)	0 (0)	2 (25)	5 (12)	2 (13)	0.18
Cough	37 (36)	5 (50)	1 (17)	3 (38)	11 (28)	3 (20)	0.53
Rhinorrhea	25 (24)	4 (40)	1 (17)	5 (62)	12 (30)	7 (47)	0.12
**Circulatory**							
Tachycardia	31 (30)	1 (10)	1 (17)	2 (25)	1 (2)	1 (7)	0.006
Cold limbs	24 (23)	4 (40)	2 (33)	2 (25)	10 (25)	5 (33)	0.84
Reduced urine output	10 (10)	0 (0)	0 (0)	0 (0)	2 (5)	0 (0)	0.82
Cyanosis	4 (4)	4 (40)	0 (0)	1 (12)	4 (10)	1 (7)	0.012
**Gastrointestinal**							
Vomiting^‡^	61 (59)	7 (70)	1 (17)	5 (62)	12 (30)	5 (33)	0.007
Constipation	34 (33)	3 (30)	0 (0)	1 (12)	3 (8)	2 (13)	0.015
Abnormal appetite	95 (91)	7 (70)	4 (67)	8 (100)	37 (92)	14 (93)	0.10
Diarrhea	10 (10)	0 (0)	0 (0)	0 (0)	2 (5)	2 (13)	0.80
**Supportive treatment**							
Mechanical ventilation	8 (8)	0 (0)	0 (0)	0 (0)	0 (0)	0 (0)	0.45
IVIG	67 (64)	5 (50)	2 (33)	3 (38)	4 (10)	6 (40)	<0.0001
Corticosteroid	92 (88)	6 (60)	4 (67)	6 (75)	20 (50)	12 (80)	0.0001
Inotropic agents	14 (13)	1 (10)	0 (0)	0 (0)	0 (0)	1 (7)	0.11

Data are n (%) or median (IQR). ^*^ The diagnoses categories were mutually exclusive. ^†^ Other neurological symptoms included nuchal rigidity, gaze palsy, nystagmus, dysarthria and reduced light reflex. The vomiting referred to non-projectile and temporary vomiting.

### Neuroimaging findings of cases with CNS complications

Magnetic resonance imaging (MRI) of the brain was performed for 41 HFMD cases with CNS complications with non-EV-A71 EVs (including CV-A6, CV-A4, CV-A2, CV-A10 and CV-A16) and 81 cases with EV-A71. Abnormal brain MRI was most frequently detected in the brainstem and cerebellum for children with non-EV-A71 EVs (S2 Table and S6 Fig), similar to the findings for those with EV-A71. Abnormal MRI lesions were also observed in other parts of the brain such as cerebral cortex for HFMD cases with CV-A10. Neuroimaging of the spine was conducted for 15 HFMD cases with CNS complications with non-EV-A71 EVs (including CV-A2, CV-A6, CV-A10 and CV-A16) and 57 cases with EV-A71. Lesions on MRI were observed in the thoracic spinal cord only in cases infected with CV-A2, while lesions were seen in both the thoracic and cervical cord in cases infected with EV-A71.

### Associations between non-CNS-specific symptoms or clinical testing indicators and diagnoses of CNS complications

**Fig 3:**
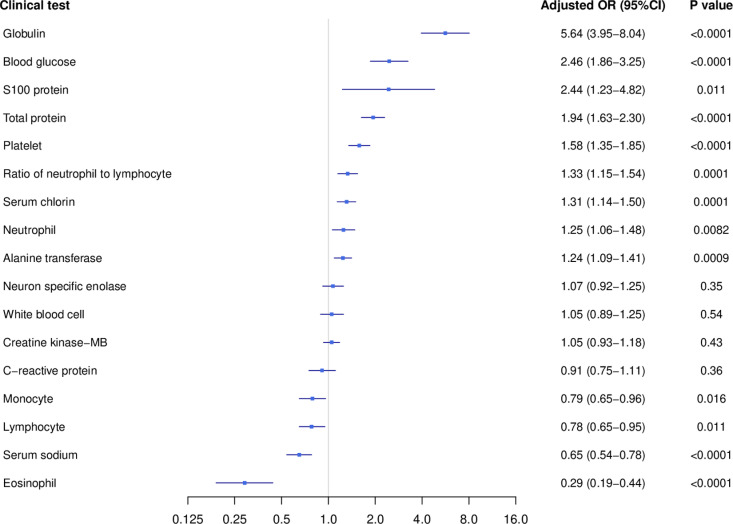
Adjusted odds ratio of standardized clinical testing indicators and CNS complications among laboratory confirmed HFMD inpatient cases controlling for controlling for age, enterovirus serotype, preterm birth, low birth weight and underlying medical conditions.

HFMD cases with CNS complications were significantly more likely to present with non-CNS-specific symptoms such as cold limbs, reduced motor activity, vomiting, fever duration ≥3 days and peak temperature ≥38.5°C; whereas they were significantly less likely to present with skin rashes and oral lesions (Table 3). Clinical testing indicators such as globulin, blood glucose, S100 protein were higher in the cases with CNS complications than in those without CNS complications with aORs significantly higher than 1 (Fig 3). In contrast, clinical testing indicators such as eosinophil counts and serum sodium were lower in the cases with CNS complications, with aORs lower than 1. The directions of association of clinical tests with CNS complications were generally similar by different serotypes and differing specimen collection time (S7–S13 Figs). Besides, aOR the neutrophil to lymphocyte ratio was 1.33 (95% CI: 1.15-1.54), slightly higher than that of neutrophil counts and the inverse of the aOR of lymphocyte counts (Fig 3).

**Table 3:** The associations between non-CNS-specific clinical manifestations and diagnoses of CNS complications among lab-confirmed inpatient HFMD cases.

Clinical manifestations	Cases without CNS Complications (N = 1538)	Cases with CNS complications (N = 230)	aOR (95% CI)^*^	P value
Cold limbs	161 (10)	61 (27)	3.58 (2.47-5.18)	<0.0001
Reduced motor activity	778 (51)	180 (78)	3.44 (2.43-4.86)	<0.0001
Vomiting^†^	292 (19)	112 (49)	3.30 (2.42-4.50)	<0.0001
Fever duration ≥ 3 days	636 (41)	172 (75)	2.88 (2.05-4.05)	<0.0001
Peak temperature ≥ 38.5°C	1288 (84)	207 (90)	1.67 (1.02-2.72)	0.041
Abnormal appetite	1314 (85)	202 (88)	1.27 (0.81-1.98)	0.30
Constipation	244 (16)	51 (22)	1.09 (0.75-1.59)	0.64
Rhinorrhea	509 (33)	71 (31)	1.01 (0.74-1.39)	0.94
Diarrhea	136 (9)	20 (9)	0.98 (0.58-1.67)	0.94
Reduced urine output	92 (6)	16 (7)	0.98 (0.54-1.77)	0.94
Cough	561 (36)	75 (33)	0.85 (0.62-1.17)	0.32
Oral lesion	1531 (100)	218 (95)	0.12 (0.04-0.35)	0.0001
Skin rash	1536 (100)	226 (98)	0.07 (0.01-0.40)	0.003

Data are n (%) or median (IQR). CNS, central nervous system; aOR, adjusted odds ratio; CI, confidence interval. ^*^aOR and 95%CI were calculated using logistic regression controlling for age, enterovirus serotype, preterm birth, low birth weight and underlying medical conditions in the multivariate analysis. ^†^ The vomiting referred to non-projectile and temporary vomiting

## Discussion

In this clinical study, we found the commonest cause of HFMD to be CV-A6, in both inpatients and outpatients. EV-A71, on the other hand, was significantly more prevalent in inpatient cases, suggesting a propensity to cause more severe disease. However, while other serotypes such as CV-A4, CV-A2, CV-A10, CV-A6 and CV-A16 in general caused mild infections, severe CNS disease did occur with all of these serotypes, which accounted for a much higher proportion of severe cases compared with prior surveillance results in Henan [9]. Whilst children with CV-A2 and CV-A4 were less likely to present with rashes on hands, feet or buttock and more likely to develop high fever, whereas those with EV-A71 were less likely to present with lesions in the mouth. HFMD cases with CNS complications with non-EV-A71 EVs were more likely to present with convulsion and less likely to present with other neurologic symptoms than those with EV-A71 with CNS disease. Several non-CNS-specific symptoms and clinical testing indicators were associated with CNS involvement.

Our findings show that EV-A71 causes more severe disease than other enterovirus serotypes, consistent with previous studies [10,11]. Moreover, our findings also suggest that patients affected by EV-A71 had higher symptom burdens, greater use of ICU, and longer hospital stays than those affected by other serotypes. Nevertheless, our study demonstrated that other non-EV-A71 EVs such as CV-A4, CV-A2, CV-A6, CV-A10 and CV-A16 could also cause severe CNS disease. Whether the damage caused non-EV-A71 EVs can lead to similar long term CNS-related sequelae as seen in children with EV-A71 remains unclear; another study has observed long term non-neurologic sequelae after CV-A6 infection, including muscle pain, joint pain, and cardiac abnormalities [12]. Of these serotypes, CV-A2 and CV-A4 are worthy of attention, because there have been few previous studies on the severity of disease caused by these two serotypes. We observed that CV-A2 and CV-A4 were over-represented in cases requiring admission to hospital, and that CV-A4 in particular, caused a significant amount of CNS disease, and even deaths as shown in another study [13]. Nevertheless, the number of patients with CNS complications was small for CV-A4 and CV-A2, and we still need to be cautious and before drawing a definitive conclusion more related studies are warranted. Given the human enteroviruses could mutate more rapidly than general RNA viruses [14,15], continuous surveillance of the causative agents of HFMD as well as their clinical manifestations is needed to detect the emergence of more virulent enteroviruses associated with severe complications.

The age range of cases hospitalized with HFMD due to EV-A71 and CV-A16 was wider compared with other serotypes, consistent with a study in another location of China [16]. This might be due to relatively stronger population immunity to EV-A71 and CV-A16 [17,18], which could be transferred to infants by mothers and therefore delaying infection with these two viruses at an earlier age compared with other new emerging serotypes [19]. The interval between illness onset and admission was longest for EV-A71, which was consistent with our observations on outpatient cases [6]. This might be because relatively more EV-A71 infected cases experience rash as the first symptom rather than fever or fever & rash, which could reduce the subjective sense of illness severity by the parents. The peak temperatures were different across serotypes and the associations between presences of rashes/ulcers and body parts also differed by serotypes to some extent. These differences between serotypes suggested it might be possible to infer the infecting serotype at population level based on clinical manifestations and is worthy of exploration in the future studies, especially for developing clinical prediction rule of EV-A71, which might be useful in primary healthcare facilities of resource-poor areas without access to diagnostic tests, or for initiating vaccine campaigns in response to outbreaks.

In our study, the presence of skin rashes and oral lesions was associated with reduced risk of CNS disease, consistent with previous reports in Vietnam, Singapore and other provinces of China, which demonstrated presences of skin rashes and oral lesions were associated with lower risks of severe disease and death [9,20]. The exanthema or oral lesions and CNS disease represent the opposite ends of the wide clinical spectrum of HFMD caused by enteroviruses. Most HFMD cases manifest as rash with oral lesions without CNS involvement [1], whereas those without rash and oral lesions may have a higher risk of developing CNS disease and warrant more careful monitoring. It is plausible for cyanosis, tachycardia, tachypnea and cold limbs to strongly associate with CNS disease, cardiopulmonary complications being another manifestation of severe enterovirus disease. Apart from fever duration of greater than three days, high fever and reduced motor activity, which have been validated in another study [21], our findings also supported vomiting was a symptom associated with CNS disease, consistent with results of a systematic review [22].

A previous systematic review reported that white blood cells (WBC) were higher in severe HFMD cases than in mild cases [23]. However, our study, which included more than twice as many patients any single study examined in the systematic review, did not find any such association, consistent with another large study that examined this relationship [24]. One explanation for this is that WBC include multiple cell types, which may have varying and opposite associations with CNS disease. Other studies used the ratio of neutrophil to lymphocyte counts as an indicator of clinical severity for both acute and chronic diseases [25,26]. Our study suggested that the ratio of neutrophil to lymphocyte counts might be a slightly more sensitive or at least equally sensitive indicator for CNS disease than separate indicators alone, because as shown in our study as well as other studies neutrophil counts and lymphocyte counts were significantly positively and negatively associated with CNS complications, respectively. This indicator is worth further verification in other clinical studies.

Both directions and strengths of the associations between clinical tests and CNS complications were examined in this study, which could provide direct evidence for selecting predictors of severe cases. Of these tests, elevated blood glucose is the indicator with a strong association with severe disease, and has been widely used for predicting clinical severity [5]. However, other routine tests such as eosinophil count and serum sodium have not been well studied in prior HFMD related research and warrant further investigation. Most of the tests in this study, except S100 protein (the sum of S100A1B and S100BB proteins) and neuron-specific enolase which were used to reveal the extent of brain damage [27,28], are routinely in inpatient paediatric practice regardless of presentation or diagnosis, and their wide availability provides the basis for their use in severity stratification of enterovirus disease in routine clinical practice.

This is one of the few comprehensive prospective HFMD case series studies characterizing clinical features and severities of not only EV-A71, CV-A16, CV-A6 and CV-A10 but also CV-A4 and CV-A2. However, this study is subject to important limitations. Firstly, the analysis of clinical manifestations was based on inpatient cases, which may not be representative of HFMD cases in outpatient and community settings. This limits the generalizability of our conclusions, and the clinical severity observed in the study population may be higher than that in the non-hospitalized population. Secondly, although this is a prospective study, more subtle symptoms present before admission were not observed by study staff and might thus be overlooked. However, parental observation of children forms the basis of paediatric history taking, so the possibility of missing significant symptoms is expected to be minimal. Thirdly, we discovered that 68% of the patients in our study originated from urban areas. This significant urban bias indicates that the study results may not accurately mirror the clinical manifestations and severity of HFMD in rural areas. Finally, our study, conducted over a one-year period at a single center, may have limitations in representing the true burden of each serotype due to potential long-term variations in their circulation. To fully understand the prevalence trends and clinical features of emerging serotypes, longer-term and multicenter studies are needed. Additionally, further research into the genetic characteristics and pathogenic mechanisms of CV-A6 is essential to facilitate the development of vaccines and treatments targeting this serotype.

In conclusion, non-EV-A71 EVs such as CV-A4, CV-A2, CV-A10, CV-A6 and CV-A16 can cause severe CNS complications. CV-A4 is clinically more severe than CV-A16, and other non-EV-A71 enteroviruses are not significantly different in term of clinical severity. CV-A6 has become the most common causative enterovirus for both inpatient and outpatient HFMD cases in central China during the study period. EV-A71 is still more likely to cause CNS disease than other enteroviruses and severe HFMD cases caused by EV-A71 also present with more severe manifestations. Continuous surveillance of emerging enteroviruses as well as their clinical severities is essential. There are non-CNS-specific symptoms as well as routine clinical tests moderately associated with CNS complications, and these clinical manifestations have potential in predicting clinical severity, and are worth attention in the clinical management of HFMD.

## Supporting information

S1 Text RT-PCR Testing at the hospital laboratory and flowchart of virological tests of throat swabs. (PDF)

S1 Table Comparison between enrolled HFMD inpatient cases and those who refused to participate in the study. (PDF)

S2 Table MRI of the brain and spine in HFMD cases with CNS complications. (PDF)

S1 Fig Weekly distribution of eligible HFMD inpatient cases who agreed and refused to participate in the study at the children’s hospital, February 2017-Februray 2018. (PDF)

S2 Fig Flowchart of enrollment of HFMD inpatient cases from February 15th 2017 to February 15th 2018 at the children’s hospital. (PDF)

S3 Fig Proportions of enterovirus serotypes among laboratory confirmed inpatient HFMD cases (N = 1768) and outpatient HFMD cases (N = 523). (PDF)

S4 Fig Time interval between illness onset and admission for enrolled HFMD inpatient cases overall and by serotype. (PDF)

S5 Fig Peak temperature across the whole disease course since illness onset by virus serotype among HFMD inpatient cases at the children’s hospital, February 2017-February 2018. (PDF)

S6 Fig MRI findings in HFMD cases with CNS complications. A) Brain MRI of cases with CV-A4 indicating lesions in dentate nucleus of the cerebellum and dorsal pons. B) Brain MRI of cases with CV-A2 indicating lesions in dentate nucleus of cerebellum and dorsal pons. C) Brain MRI of cases with CV-A10 indicating cerebral cortex involvement. D) Brain MRI of cases with CV-A6 indicating lesions in dorsal pons. E) Brain MRI of cases with CV-A16 indicating lesions in dorsal pons and medulla oblongata. F) Spine MRI of cases with CV-A6 indicating lesions in thoracic spine.
(PDF)

S7 Fig Associations of complete blood count with CNS complications by specimen collection time among laboratory confirmed HFMD inpatient cases. A) White blood cell. B) Neutrophil. C) Lymphocyte. D) Monocyte. E) Eosinophil. F) Platelet. The red asterisks indicate statistical significance.
(PDF)

S8 Fig Associations of complete blood count with CNS complications by virus serotype among laboratory confirmed HFMD inpatient cases. A) White blood cell. B) Neutrophil. C) Lymphocyte. D) Monocyte. E) Eosinophil. F) Platelet. The red asterisks indicate statistical significance.
(PDF)

S9 Fig Associations of neurological injury tests, myocardial enzymes and electrolytes with CNS complications by specimen collection time among laboratory confirmed HFMD inpatient cases. A) S100 protein. B) Neuron−specific enolase. C) Creatine kinase MB. D) Alanine transferase. E) Serum sodium. F) Serum chloride. The red asterisks indicate statistical significance.
(PDF)

S10 Fig Associations of neurological injury tests, myocardial enzymes and electrolytes with CNS complications by virus serotype among laboratory confirmed HFMD inpatient cases. A) S100 protein. B) Neuron−specific enolase. C) Creatine kinase MB. D) Alanine transferase. E) Serum sodium. F) Serum chloride. The red asterisks indicate statistical significance.
(PDF)

S11 Fig Associations of blood proteins and glucose with CNS complications by specimen collection time among laboratory confirmed HFMD inpatient cases. A) Total protein. B) Globulin. C) C-reactive protein. D) Blood glucose. The red asterisks indicate statistical significance.
(PDF)

S12 Fig Associations of blood proteins and glucose with CNS complications by virus serotype among laboratory confirmed HFMD inpatient cases. A) Total protein. B) Globulin. C) C-reactive protein. D) Blood glucose. The red asterisks indicate statistical significance.
(PDF)

S13 Fig Associations between results of the main clinical tests with presence of CNS complications among laboratory confirmed HFMD inpatient cases. A-F) Complete blood count (A, white blood cell; B, neutrophil; C, lymphocyte; D, monocyte; E, eosinophil; F, platelet). G-H) Neurological injury tests (G, S100 protein; H, neuron−specific enolase). I-J) Myocardial enzymes (I, creatine kinase MB; J, alanine transferase). K-L) Electrolytes (K, serum sodium; L, serum chloride). M-P) Blood proteins and glucose (M, total protein; N, globulin; O, C-reactive protein; P, blood glucose). The red asterisks indicate statistical significance.
(PDF)

S1 File Data used to obtain Figs 1–3, S1, S3-S5 and S7-S13. (XLSX)
