# Supplementary material for: Clinical characteristics and severity of hand, foot, and mouth disease by virus serotype: A prospective hospital-based cohort study
Source: PLoS Negl Trop Dis. 2025 May 23;19(5):e0013039. doi: 10.1371/journal.pntd.0013039 (PMC12101662; doi:10.1371/journal.pntd.0013039)
Supplement: S1 Text — (PDF) [file pntd.0013039.s001.pdf]

S1 Text. RT-PCR Testing at the hospital laboratory and flowchart of virological tests of throat swabs

The commercial one-step multiplex RT-PCR kit (EV-A71, CV-A16 and other enterovirus Viral RNA Qualitative Diagnostic Kit (PCR Fluorescence Probing), mole, China, patent number: 20143402367), which were used for etiological testing at the hospital laboratory, consists of extract kit (A) and one-step RT-PCR kit (B). Briefly, viral RNA was extracted based on the column-centrifugation method and amplified using generic (pan-enterovirus) and specific (EV-A71 and CV-A16) primers and probes per manufacturer protocol. The reactions of the real time RT-PCR were run under the following conditions: incubation at 45 °C for 15 min, initial denaturation at 95°C for 2 min, followed by 40 cycles of denaturation at 94 °C for 10 s, annealing and extension at 58 °C for 40 s.

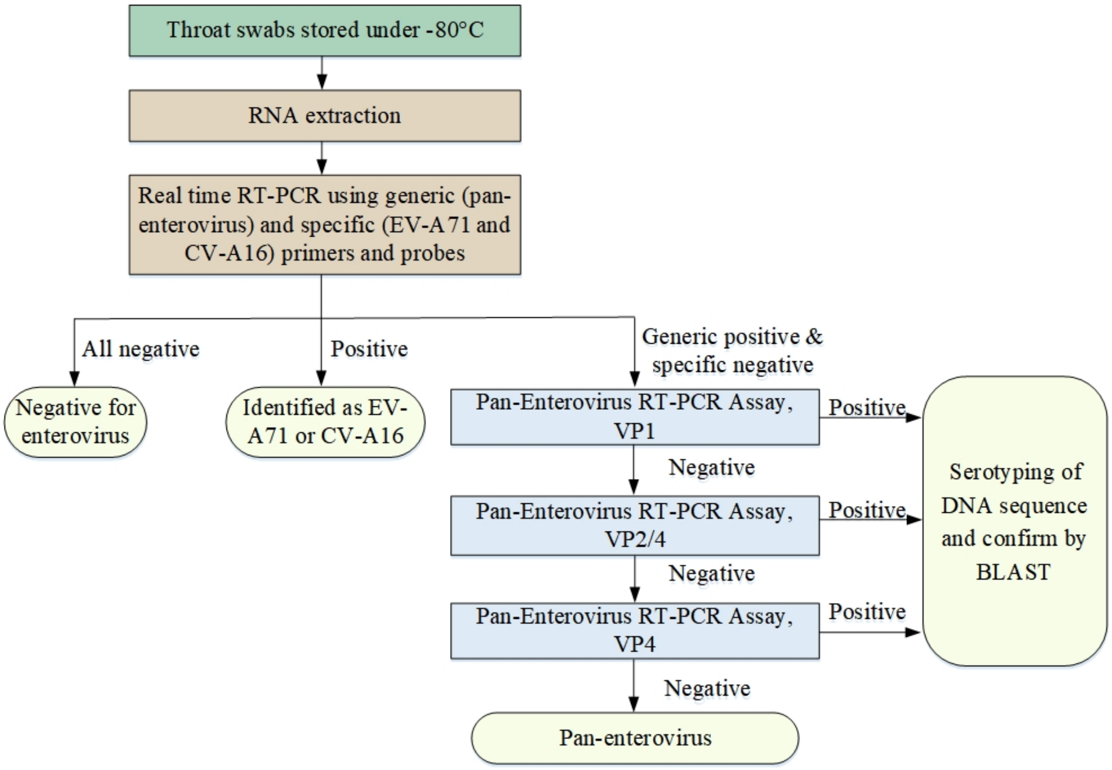

Flowchart of virological tests of throat swabs.
