# Supplementary material for: Clinical characteristics and severity of hand, foot, and mouth disease by virus serotype: A prospective hospital-based cohort study
Source: PLoS Negl Trop Dis. 2025 May 23;19(5):e0013039. doi: 10.1371/journal.pntd.0013039 (PMC12101662; doi:10.1371/journal.pntd.0013039)
Supplement: S1 Table — (PDF) [file pntd.0013039.s002.pdf]

**S1 Table. Comparison between enrolled HFMD inpatient cases and those who refused to participate in the study**

| Characteristics | Enrolled (N=1840) | Refused (N=684) | P |
|---|---|---|---|
| **Male** | 1162 (63) | 410 (60) | 0.15 |
| **Age, years** | | | |
| Mean | 2.09 | 2.10 | 0.77 |
| Median | 1.66 | 1.69 | 0.78 |
| **Intensive care unit admission** | 73 (4) | 1 (0) | <0.0001 |

Data are n (%).
