# Supplementary material for: Clinical characteristics and severity of hand, foot, and mouth disease by virus serotype: A prospective hospital-based cohort study
Source: PLoS Negl Trop Dis. 2025 May 23;19(5):e0013039. doi: 10.1371/journal.pntd.0013039 (PMC12101662; doi:10.1371/journal.pntd.0013039)
Supplement: S2 Table — (PDF) [file pntd.0013039.s003.pdf]

S2 Table. MRI of the brain and spine in HFMD cases with CNS complications

Lesion location in MRI	EV-A71 (N=104)	CV-A4 (N=10)	CV-A2 (N=6)	CV-A10 (N=8)	CV-A6 (N=40)	CV-A16 (N=15)
**Number of cases receiving brain MRI**	81 (78)	8 (80)	3 (50)	6 (75)	17 (42)	7 (47)
Brainstem	41 (51)	1 (12)	3 (100)	0 (0)	1 (6)	1 (14)
Cerebellum	34 (42)	1 (12)	3 (100)	0 (0)	1 (6)	0 (0)
Thalamus	1 (1)	0 (0)	0 (0)	0 (0)	0 (0)	0 (0)
Basal nucleus	1 (1)	0 (0)	0 (0)	0 (0)	0 (0)	0 (0)
Lateral ventricle	3 (4)	1 (12)	0 (0)	1 (17)	0 (0)	0 (0)
Cerebral cortex	2 (2)	0 (0)	0 (0)	1 (17)	0 (0)	0 (0)
Subcortex White matter	3 (4)	0 (0)	0 (0)	1 (17)	0 (0)	1 (14)
**Number of receiving spinal cord MRI**	57 (55)	0 (0)	1 (17)	2 (25)	9 (23)	3 (20)
Cervical spine	14 (25)	NA	0 (0)	1 (50)	0 (0)	0 (0)
Thoracal spine	15 (26)	NA	1 (100)	1 (50)	2 (22)	0 (0)
Lumbar spine	8 (14)	NA	0 (0)	0 (0)	1 (11)	0 (0)

Data are n (%).
