# Supplementary material for: Clinical characteristics and severity of hand, foot, and mouth disease by virus serotype: A prospective hospital-based cohort study
Source: PLoS Negl Trop Dis. 2025 May 23;19(5):e0013039. doi: 10.1371/journal.pntd.0013039 (PMC12101662; doi:10.1371/journal.pntd.0013039)
Supplement: S3 Fig — (PDF) [file pntd.0013039.s006.pdf]

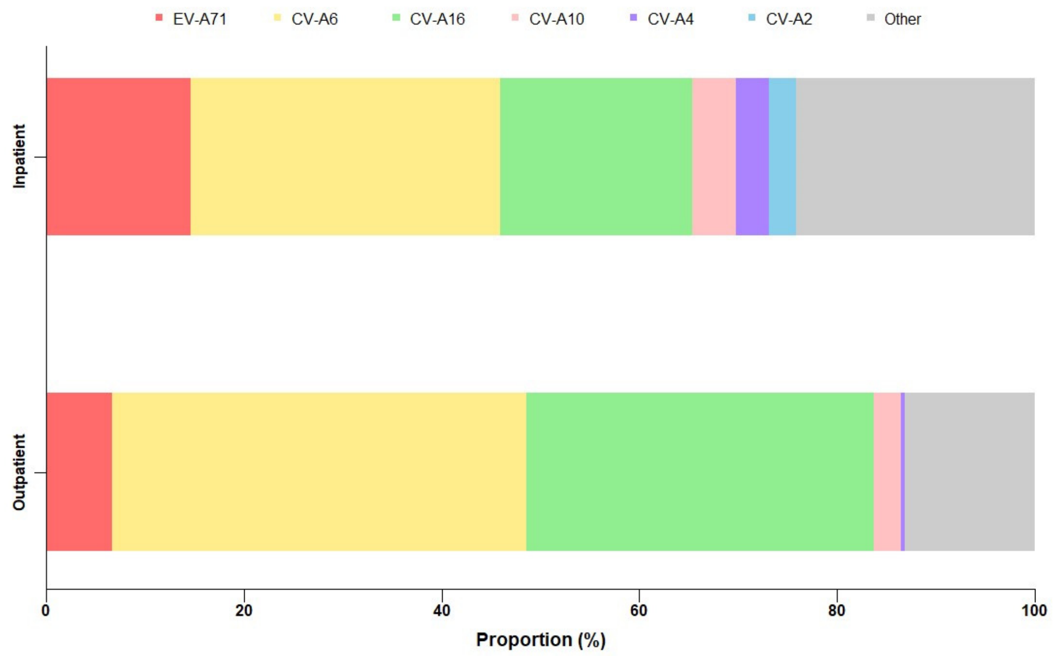

S3 Fig. Proportions of enterovirus serotypes among laboratory confirmed inpatient HFMD cases (N=1768) and outpatient HFMD cases (N=523).
