# Supplementary material for: Clinical characteristics and severity of hand, foot, and mouth disease by virus serotype: A prospective hospital-based cohort study
Source: PLoS Negl Trop Dis. 2025 May 23;19(5):e0013039. doi: 10.1371/journal.pntd.0013039 (PMC12101662; doi:10.1371/journal.pntd.0013039)
Supplement: S4 Fig — (PDF) [file pntd.0013039.s007.pdf]

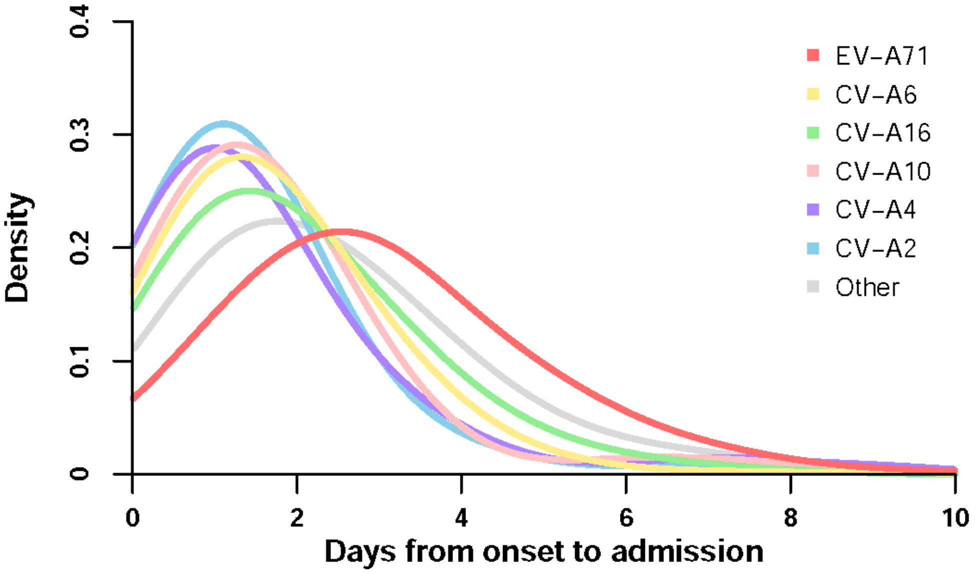

S4 Fig. Time interval between illness onset and admission for enrolled HFMD inpatient cases overall and by serotype.
