# Supplementary material for: Clinical characteristics and severity of hand, foot, and mouth disease by virus serotype: A prospective hospital-based cohort study
Source: PLoS Negl Trop Dis. 2025 May 23;19(5):e0013039. doi: 10.1371/journal.pntd.0013039 (PMC12101662; doi:10.1371/journal.pntd.0013039)
Supplement: S5 Fig — (PDF) [file pntd.0013039.s008.pdf]

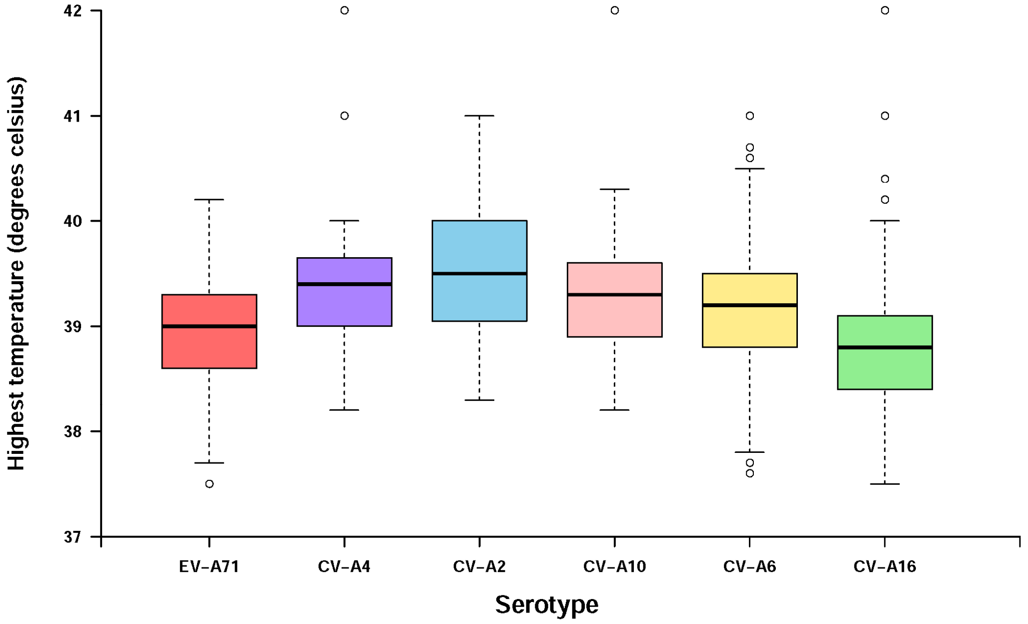

S5 Fig. Peak temperature across the whole disease course since illness onset by virus serotype among HFMD inpatient cases at the children's hospital, February 2017-February 2018.
