# Supplementary material for: Clinical characteristics and severity of hand, foot, and mouth disease by virus serotype: A prospective hospital-based cohort study
Source: PLoS Negl Trop Dis. 2025 May 23;19(5):e0013039. doi: 10.1371/journal.pntd.0013039 (PMC12101662; doi:10.1371/journal.pntd.0013039)
Supplement: S6 Fig — A) Brain MRI of cases with CV-A4 indicating lesions in dentate nucleus of the cerebellum and dorsal pons. B) Brain MRI of cases with CV-A2 indicating lesions in dentate nucleus of cerebellum and dorsal pons. C) Brain MRI of cases with CV-A10 indicating cerebral cortex involvement. D) Brain MRI of cases with CV-A6 indicating lesions in dorsal pons. E) Brain MRI of cases with CV-A16 indicating lesions in dorsal pons and medulla oblongata. F) Spine MRI of cases with CV-A6 indicating lesions in thoracic spine. (PDF) [file pntd.0013039.s009.pdf]

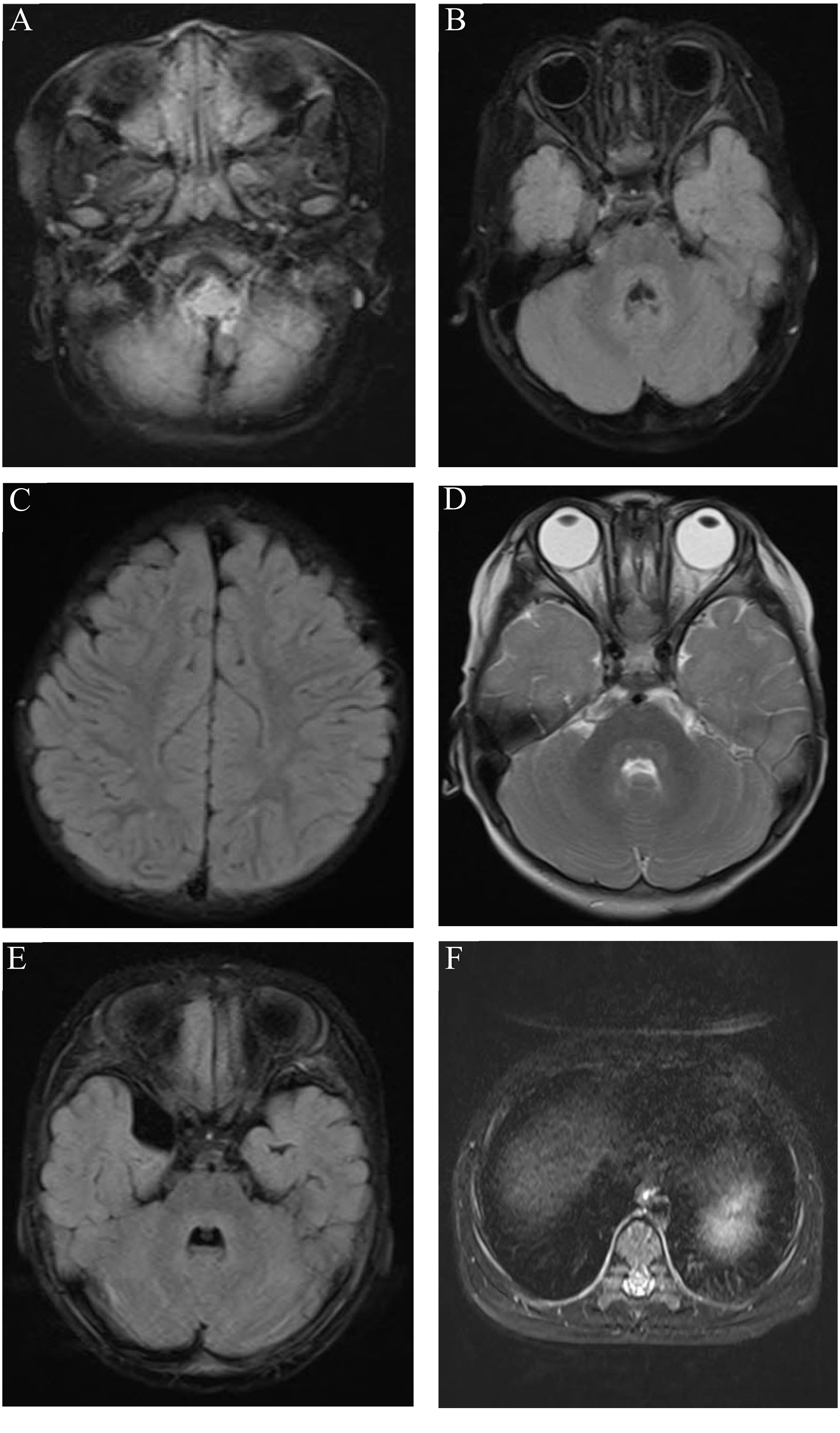
A
B
C
D
E
F

S6 Fig. MRI findings in HFMD cases with CNS complications. A) Brain MRI of cases with CV-A4 indicating lesions in dentate nucleus of the cerebellum and dorsal pons. B) Brain MRI of cases with CV-A2 indicating lesions in dentate nucleus of cerebellum and dorsal pons. C) Brain MRI of cases with CV-A10 indicating cerebral cortex involvement. D) Brain MRI of cases with CV-A6 indicating lesions in dorsal pons. E) Brain MRI of cases with CV-A16 indicating lesions in dorsal pons and medulla oblongata. F) Spine MRI of cases with CV-A6 indicating lesions in thoracic spine.
