# Supplementary material for: Clinical characteristics and severity of hand, foot, and mouth disease by virus serotype: A prospective hospital-based cohort study
Source: PLoS Negl Trop Dis. 2025 May 23;19(5):e0013039. doi: 10.1371/journal.pntd.0013039 (PMC12101662; doi:10.1371/journal.pntd.0013039)
Supplement: S7 Fig — A) White blood cell. B) Neutrophil. C) Lymphocyte. D) Monocyte. E) Eosinophil. F) Platelet. The red asterisks indicate statistical significance. (PDF) [file pntd.0013039.s010.pdf]

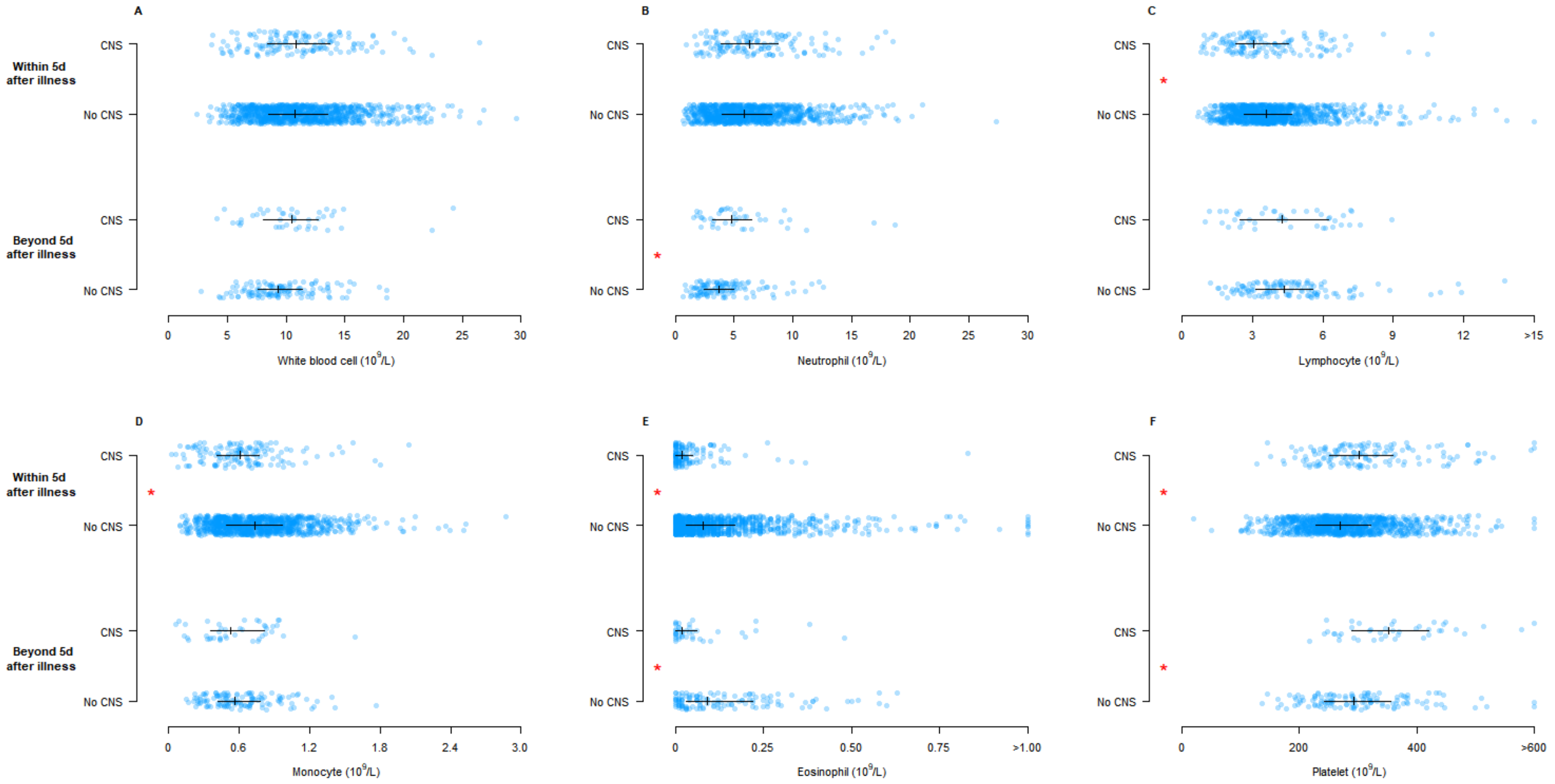

S7 Fig. Associations of complete blood count with CNS complications by specimen collection time among laboratory confirmed HFMD inpatient cases. A) White blood cell. B) Neutrophil. C) Lymphocyte. D) Monocyte. E) Eosinophil. F) Platelet. The red asterisks indicate statistical significance.
