# Supplementary material for: Clinical characteristics and severity of hand, foot, and mouth disease by virus serotype: A prospective hospital-based cohort study
Source: PLoS Negl Trop Dis. 2025 May 23;19(5):e0013039. doi: 10.1371/journal.pntd.0013039 (PMC12101662; doi:10.1371/journal.pntd.0013039)
Supplement: S9 Fig — A) S100 protein. B) Neuron−specific enolase. C) Creatine kinase MB. D) Alanine transferase. E) Serum sodium. F) Serum chloride. The red asterisks indicate statistical significance. (PDF) [file pntd.0013039.s012.pdf]

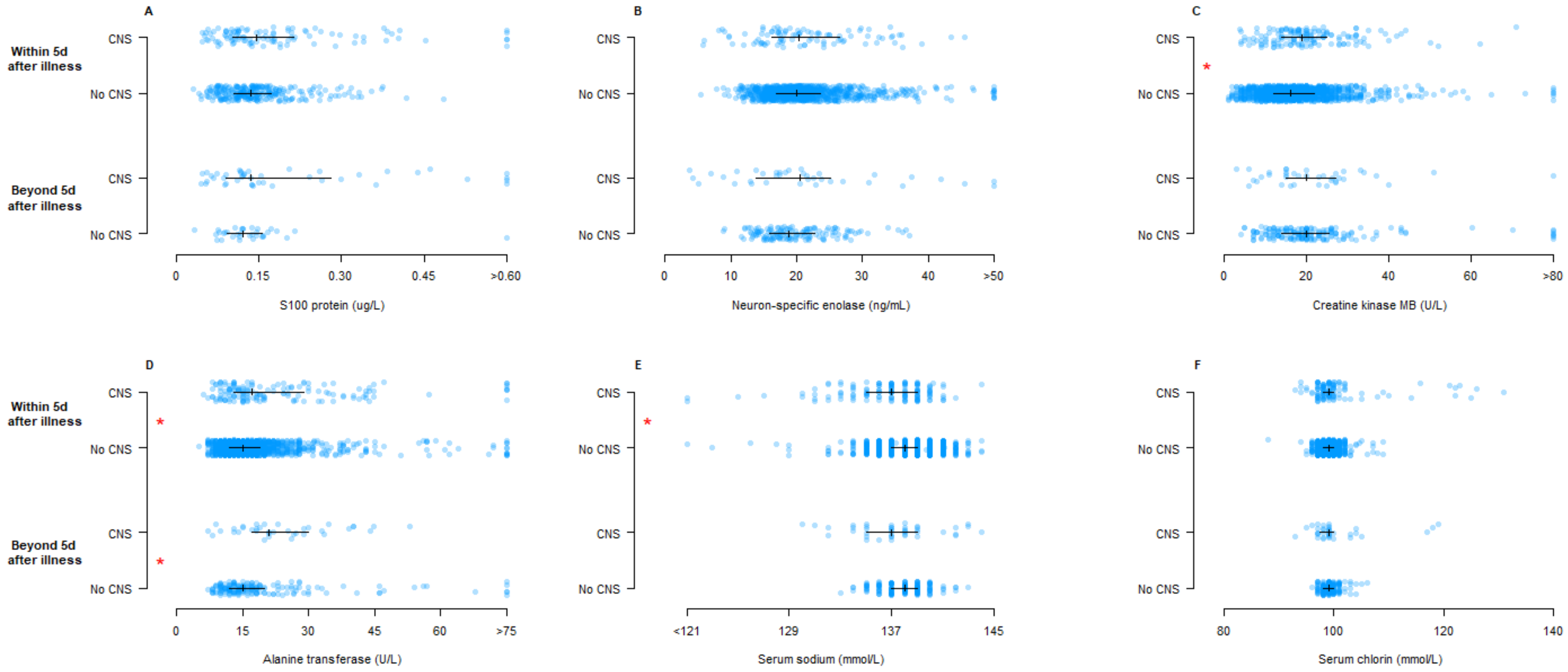

S9 Fig. Associations of neurological injury tests, myocardial enzymes and electrolytes with CNS complications by specimen collection time among laboratory confirmed HFMD inpatient cases. A) S100 protein. B) Neuron−specific enolase. C) Creatine kinase MB. D) Alanine transferase. E) Serum sodium. F) Serum chloride. The red asterisks indicate statistical significance.
