# Supplementary material for: Clinical characteristics and severity of hand, foot, and mouth disease by virus serotype: A prospective hospital-based cohort study
Source: PLoS Negl Trop Dis. 2025 May 23;19(5):e0013039. doi: 10.1371/journal.pntd.0013039 (PMC12101662; doi:10.1371/journal.pntd.0013039)
Supplement: S11 Fig — A) Total protein. B) Globulin. C) C-reactive protein. D) Blood glucose. The red asterisks indicate statistical significance. (PDF) [file pntd.0013039.s014.pdf]

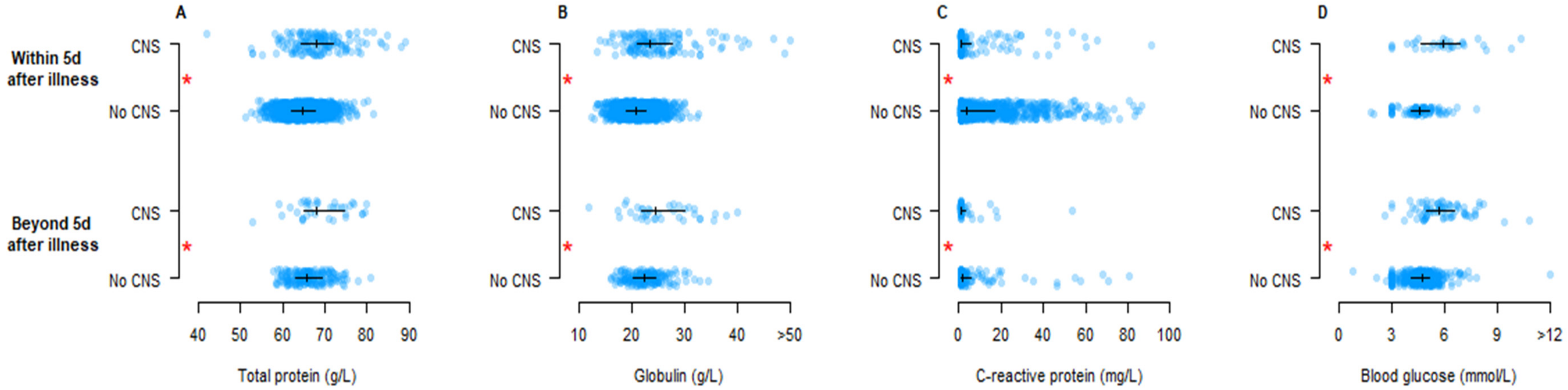

S11 Fig. Associations of blood proteins and glucose with CNS complications by specimen collection time among laboratory confirmed HFMD inpatient cases. A) Total protein. B) Globulin. C) C-reactive protein. D) Blood glucose. The red asterisks indicate statistical significance.
