# Supplementary material for: Clinical characteristics and severity of hand, foot, and mouth disease by virus serotype: A prospective hospital-based cohort study
Source: PLoS Negl Trop Dis. 2025 May 23;19(5):e0013039. doi: 10.1371/journal.pntd.0013039 (PMC12101662; doi:10.1371/journal.pntd.0013039)
Supplement: S13 Fig — A-F) Complete blood count (A, white blood cell; B, neutrophil; C, lymphocyte; D, monocyte; E, eosinophil; F, platelet). G-H) Neurological injury tests (G, S100 protein; H, neuron−specific enolase). I-J) Myocardial enzymes (I, creatine kinase MB; J, alanine transferase). K-L) Electrolytes (K, serum sodium; L, serum chloride). M-P) Blood proteins and glucose (M, total protein; N, globulin; O, C-reactive protein; P, blood glucose). The red asterisks indicate statistical significance. (PDF) [file pntd.0013039.s016.pdf]

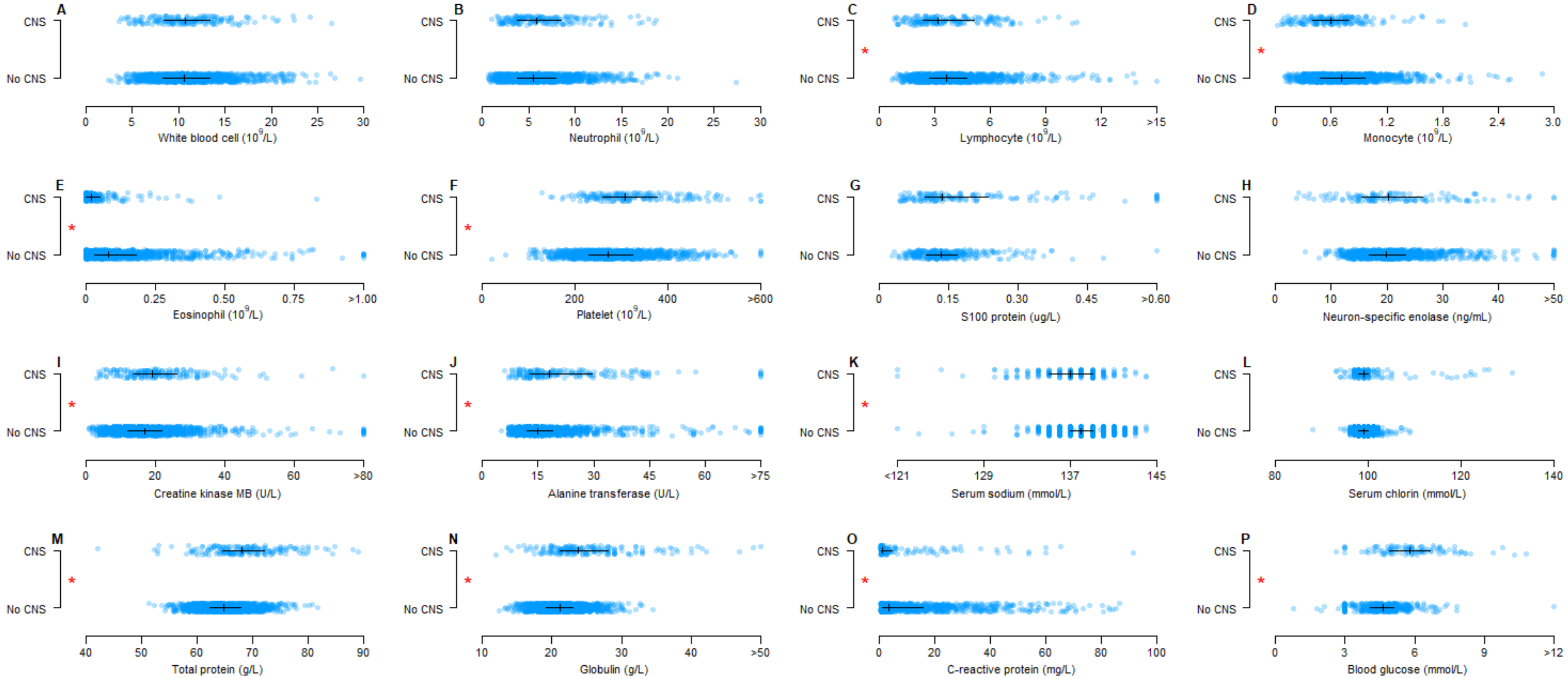

S13 Fig. Associations between results of the main clinical tests with presence of CNS complications among laboratory confirmed HFMD inpatient cases. A-F) Complete blood count (A, white blood cell; B, neutrophil; C, lymphocyte; D, monocyte; E, eosinophil; F, platelet). G-H) Neurological injury tests (G, S100 protein; H, neuron−specific enolase). I-J) Myocardial enzymes (I, creatine kinase MB; J, alanine transferase). K-L) Electrolytes (K, serum sodium; L, serum chloride). M-P) Blood proteins and glucose (M, total protein; N, globulin; O, C-reactive protein; P, blood glucose). The red asterisks indicate statistical significance.
